# *In vitro* effects of the small-molecule protein kinase C agonists on HIV latency reactivation

**DOI:** 10.1038/srep39032

**Published:** 2016-12-12

**Authors:** Jessica Brogdon, Widade Ziani, Xiaolei Wang, Ronald S. Veazey, Huanbin Xu

**Affiliations:** 1 Tulane National Primate Research Center, Tulane University School of Medicine, Covington, LA 70433, USA; 2 Tulane National Primate Research Center, Pathology and Laboratory Medicine, Tulane University School of Medicine, Covington, LA 70433, USA

## Abstract

The persistence of latently HIV-infected cellular reservoirs represents the major obstacle to virus eradication in patients under antiretroviral therapy (ART). Cure strategies to eliminate these reservoirs are thus needed to reactivate proviral gene expression in latently infected cells. In this study, we tested optimal concentrations of PKC agonist candidates (PEP005/Ingenol-3-angelate, prostratin, bryostatin-1, and JQ1) to reactivate HIV latency *in vitro*, and examined their effects on cell survival, activation and epigenetic histone methylation after treatment alone or in combination in cell line and isolated CD4 T cells from SIV-infected macaques. The results showed that PKC agonists increased cell activation with different degrees of latency reactivation, concomitant with reduced levels of histone methylation. With increasing concentrations, prostratin and byrostain-1 treatment rapidly reduced cell survival and cell activation. The PKC agonist combinations, or in combination with JQ1, led to modest levels of synergistic reactivation of HIV. Remarkably, PEP005 treatment alone caused marked reactivation of HIV latency, similar to PMA stimulation. These findings suggested that PEP005 alone, as indicated its lower cytotoxicity and lower effective dose inducing maximal reactivation, might be a candidate for effectively reactivating HIV latency as part of a therapeutic strategy for HIV infection.

Antiretroviral therapy (ART), using a combination of three or more antiretroviral drugs, has dramatically reduced HIV-1 replication and viremia below the clinical limit of detection and prevents the rate of progression to AIDS. However, residual low-level replication-competent HIV-1 still persists in a latent state in the form of integrated and transcriptionally silent proviruses. Long-lived viral reservoirs are unaffected by long-term ART or host immune responses, resulting in lifelong infection and viral rebound to pre-treatment levels in the vast majority of HIV-1-infected individuals when ART is discontinued^1^,^2^,^3^,^4^,^5^,^6^,^7^. Lymphoid tissues such as tonsil and lymph node have been described as the major sites for HIV persistence, replication and latency^8^,^9^. The persistence of latent HIV-infected cellular reservoirs represents the major hurdle to virus eradication in patients treated with ART. The stability of HIV reservoirs is consistent with long-term survival of naïve, resting memory CD4+ cells (over 20 years). Since the transcription of HIV genes depends on the activation state of cells, the integrated HIV DNA is transcriptionally silent in these cells, and therefore unaffected by ART^10^. Thus, the “shock and kill” strategy has been proposed to antagonize HIV-1 latency in viral reservoirs by several therapeutic agents in combination with ART. Recently, doctors and scientists in the UK report that new pioneering anti-retroviral treatment in HIV+ patients, in combination with boosted immune responses and a latent reversing agent (LRA)-vorinostat called the “kick and kill” strategy, may have worked in an HIV patient, as indicated undetectable viral load in blood, albeit it is uncertain whether this patient has truly been cured (http://www.iflscience.com/health-and-medicine/a-british-man-may-have-been-cured-of-hiv/). Cells harboring latent HIV provirus are activated by cytokines (e.g., IL-2)^11^,^12^, lipopolysaccharides^13^, bacterial superantigens^14^, anti-T cell antibodies (OKT3)^15^, and small-molecule LRAs including histone deacetylase inhibitors and Protein Kinase C (PKC) agonists^16^. Upon reactivated by LRAs, the virus latently infected cells could be eliminated though viral cytopathic effects or host cytolytic T lymphocyte (CTL) responses^17^,^18^,^19^. Histone modification contributes to regulation of active gene expression and latency reactivation. For examples, histone acetylation is associated with increased transcription while deacetylation induces gene repression. Histone deacetylase inhibitors (HDACi), including suberoylanilide hydroxamic acid (SAHA; Vorinostat), romidepsin, and panobinostat, are thus used to activate viral latency or cancer cells through suppression of histone deacetylases that enzymatically remove the acetyl group from histones^20^,^21^,^22^,^23^,^24^. In contrast to HDACi, natural or semisynthetic protein kinase C (PKC) activators, could strongly reactivate HIV in cell line models and primary CD4+ T cells without inducing tumor formation^17^,^25^,^26^,^27^. The PKC pathway plays an important role in cellular latency reactivation via a NF-κB signaling as well as by a positive transcription elongation factor b (P-TEFb)-dependent manner. Various small-molecule PKC activators, including PEP005, prostratin and bryostatin-1, are helpful to reactivate HIV latency. PEP005 (ingenol-3-angelate), an activator of protein kinase C (PKC), induces nuclear translocation of PKCδ, exhibiting activity of clinical anti-tumor and actinic keratosis^28^,^29^,^30^,^31^, and potential for HIV latency reactivation^32^,^33^,^34^. Prostratin is another PKC activator that extracted from the tropical plant, *Homalanthus nutans* with potent anti-tumor and cell activation properties. Prostratin not only induces HIV expression from latently infected cells through phosphorylation and degradation of IκBα, leading to the rapid nuclear translocation of NF-κB^35^, but also inhibits HIV entry by interacting with a cellular target necessary for viral entry, displaying potent antiviral activity against different strains of HIV-1 and SIV^36^,^37^,^38^,^39^. Likewise, PKC agonist bryostatin-1, isolated from the marine invertebrate *Bugula neritina*^40^,^41^, also shows excellent potential as a therapeutic agent that acts through modulation of PKC signal transduction, as indicated its potency to revert HIV-1 latency via the adenosine monophospate (AMP)-activated protein kinase (AMPK) pathway^25^,^42^,^43^,^44^,^45^,^46^. However, recent study of bryostatin-1 treatment shows inhibitory effects on the HIV-specific CD8+ T cells^47^. To fully enhance reactivation of HIV latency, these LRAs, their combination or in combination with others, are examined in cell line models *in vitro*^48^. JQ1 is another small molecule inhibitor of the bromodomain and extra-terminal (BET) family of bromodomain proteins with high affinity for BRD4 that associates with acetylated chromatin for active transcription. JQ1 competitively binds to BRD4, thereby preventing BRD4 from binding to positive transcription elongation factor b (P-TEFb)^49^. Since JQ1 displaces P-TEFb from BRD4, resulting in formation of P-TEFb/HIV Tat complexes and subsequent recruitment to the HIV LTR, JQ1 is thus believed to specifically initiate HIV gene expression in HIV-infected cells^50^,^51^. Therefore, it is valuable to seek compounds suitable for HIV reactivation from reservoirs with low or minimal cytotoxicity, especially for assessing their potential for therapeutic applications *in vivo*.

In this study, we tested various concentrations of Protein kinase C (PKC) agonists (PEP005/Ingenol-3-angelate, prostratin and bryostatin-1) for their reactivation potential in HIV latency cell models *in vitro*, and compared their effects on cell survival, activation and histone methylation after treatment alone, in combination, or in combination with JQ1. Effects of optimized PKC agonist treatment alone or combination on the reactivation of primary CD4 T cell isolated from SIV-infected macaques were also evaluated.

## Results

### Optimal dosing for maximal effects of small-molecule compounds, and their combination on reactivation of HIV latency *in vitro*

**Figure 1:**
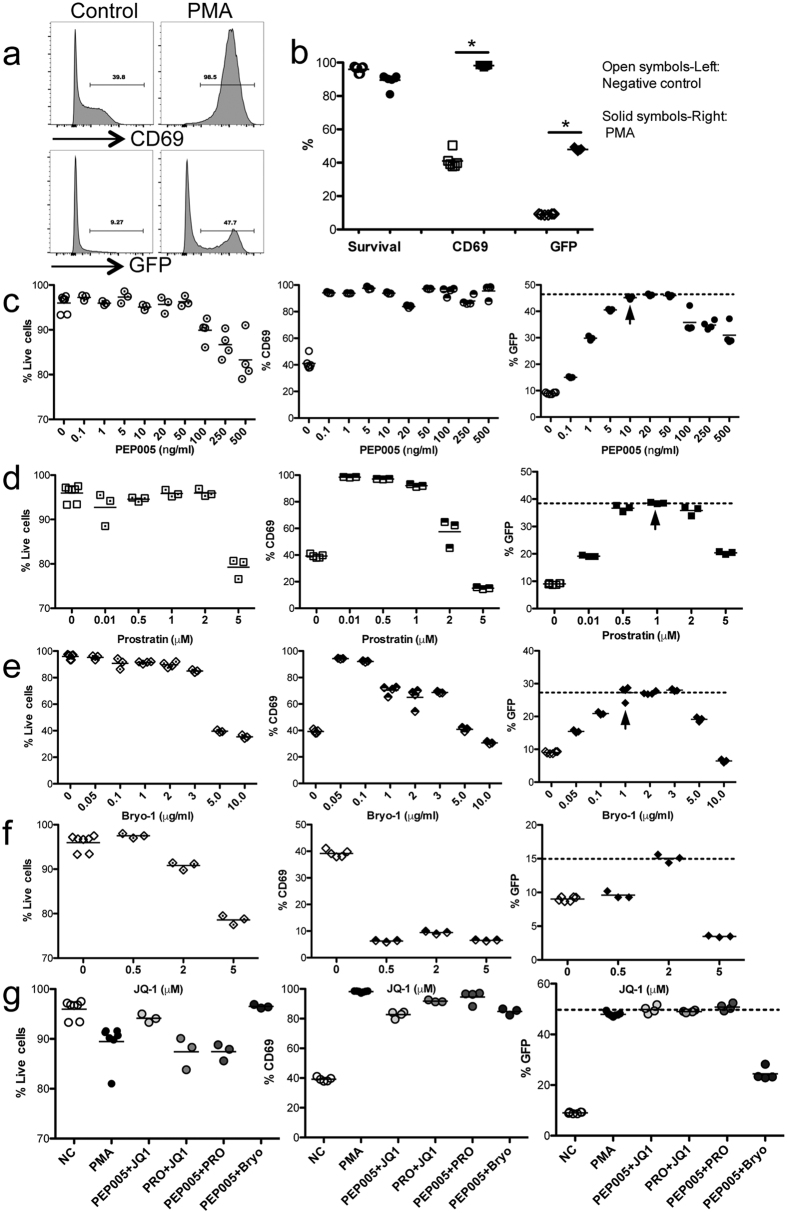
Small-molecule compounds reactivate HIV latency in a dose-dependent manner in J-Lat-Tat-GFP cells *in vitro*. (**a**) Representative histogram of CD69 and GFP expression in cells in the presence or absence of PMA stimulation for 24 hrs; (**b**) Levels of cell survival, CD69 and GFP expression in control or PMA stimulated cells. *P < 0.05. The small-molecule compounds include PEP005 (**c**), prostratin (**d**) and bryostatin-1 (**e**), and JQ1 (**f**). Cells (5 × 10^5^/well) were cultured in the presence of different concentration of each compound for 24 hrs. Cell survival, the early cell activation marker CD69, and GFP expression were analyzed. Note the effects of PEP005 treatment alone on HIV latency reactivation were identical to that of PMA stimulation. (**g**) The levels of cell survival, CD69 and GFP expression in presence of optimal concentration of PKC activators in combination, with or without JQ1. These data are representative of at least three independent experiments.

The J-Lat-Tat-GFP cell line is derived from Jurkat T lymphocytes, which bear the integrated HIV LTR-Tat and GFP gene^52^,^53^,^54^. Resting cells are GFP negative, but cell activation leads to HIV-1 LTR-driven GFP expression. As indicated in Fig. 1a and b, after PMA (final 5 ng/ml) stimulation for 24 h, higher percentage of cells (~45%) expressed GFP, compared with unstimulated controls (~5%). In addition to GFP expression, cells stimulated by PMA also significantly increased early cell activation (CD69). These results indicate the J-Lat-Tat-GFP cell line represents an appropriate cell model to evaluate reactivation of HIV latency *in vitro*. To determine the optimal concentration of each PKC agonist and JQ1 in inducing HIV latency reversal, cells were cultured in the presence of a varying concentrations of each compound for 24 h, then harvested to detect cell survival, GFP and CD69 expression. The data show PKC agonists displayed different concentration-dependent effects on cell activation and GFP expression. Optimal concentrations accompanied by >90% cell survival were as follows: PEP005 (10 ng/ml; GFP, ~45%; CD69, ~94%); prostratin (1 μM or 390 μg/ml; GFP, ~38.5%; CD69, ~92%); and bryostatin-1 (1 μg/ml; GFP, ~28%; CD69, ~70%). At these concentrations, PEP005 treatment showed the maximal potential HIV latency reactivation as indicated by GFP expression (~45%), followed by prostratin (~38.5%), and the lowest was bryostatin-1 (~28%). With increasing concentrations, GFP expression was rapidly reduced in prostratin treated (from 1 μM to 5 μM) or byrostatin-1 treated (from 1 μg/ml to 10 μg/ml) cells, concomitant with reduced cell survival at the highest concentrations tested (<80% or 40%, respectively), compared with a broad range of tolerance to PEP005 treatment (from 10 ng/ml to 500 ng/ml, with cell survival >80% even at the highest concentration) (Fig. 1c–e).

We also tested the bromodomain inhibitor JQ1 that has been previously shown to synergize with PEP005 or prostratin to re-activate latently infected cells *in vitro*^32^,^51^. JQ1 is a small molecule inhibitor of the bromodomain and extra-terminal (BET) family of bromodomain proteins with the high affinity for BRD4. BRD4 is associates with acetylated chromatin for active transcription. JQ1 competitively binds to BRD4, thereby preventing BRD4 from binding to positive transcription elongation factor b (P-TEFb)^49^. Here we tested the effects of JQ1 treatment alone for its capacity to induce HIV latency reactivation, as indicated in Fig. 1f. However, note only ~15% cells expressed GFP after JQ1 treatment. PEP005 treatment alone induced identical cell reactivation levels to those of PMA, and significantly higher levels of cell reactivation than JQ1, prostratin, bryostatin-1 or SAHA (data not shown) treatments alone. Combined, these findings indicated that of the drugs tested alone, PEP005 was the best candidate to efficiently reactivate HIV latency by inducing the highest levels of cell activation and latency reversal but with the lowest cytotoxicity.

**Figure 2:**
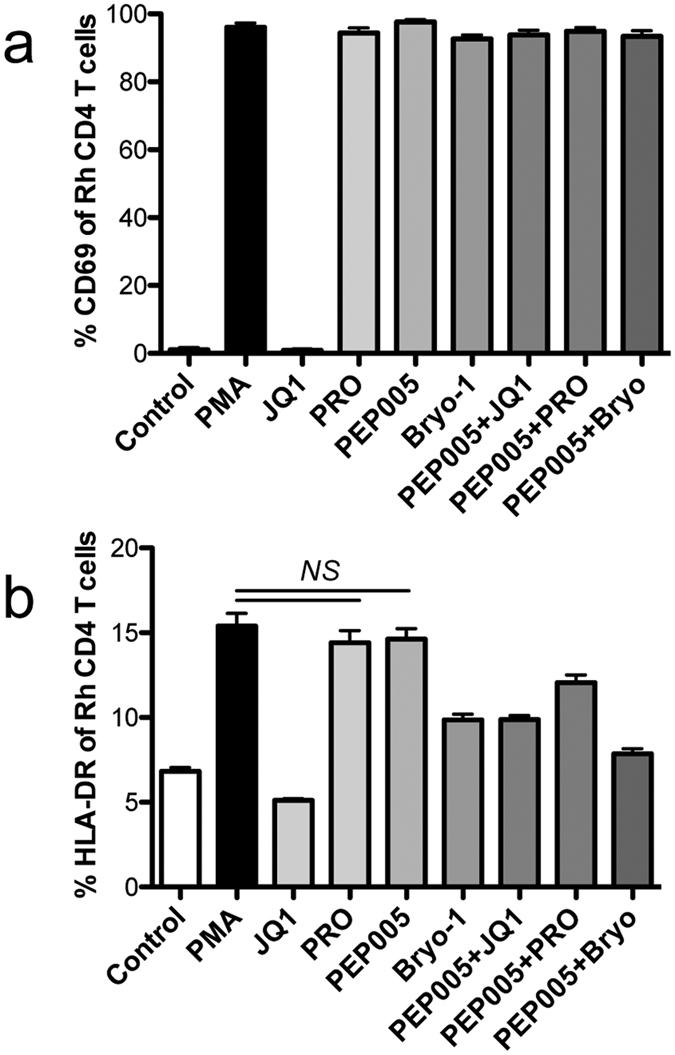
Effects of compound treatment alone or/and combination on HIV latency reactivation in primary CD4 T cells from SIV-infected rhesus macaques *in vitro*. CD4 T cell activation (CD69, (**a**) or HLA-DR, (**b**) were analyzed before and after stimulation for 24 h. CD4 T cells were negatively isolateed by microbeads. These data are representative of at least three independent experiments.

Recent reports indicate that PKC agonists, in combination with JQ1, exhibit synergism in reactivation of latent HIV^32^,^45^. In this study, PKC agonists in combination, and with JQ1 were evaluated for their potential to reactivate HIV latency. Using the combination of optimal concentration of each compound, our data showed that combinations of PEP005+JQ1, prostratin+JQ1, or PEP005+prostratin, synergistically induced HIV latency reactivation to some extent, but the PEP005+bryostatin-1 combination did not (Fig. 1g). Consistent with results in J-Lat cell lines, treatment with PKC agonist alone or in combination also showed activation of primary CD4 T cells isolated from rhesus macaques, as indicated by significant increases of CD69 and HLA-DR expression. However, PEP005, in combination with prostratin, bryotatin-1 or JQ1, did not increase cell activation, compared with PEP005 alone (Fig. 2a and b). These results suggested that PEP005 alone may be enough to efficiently reactivate HIV latency as combinations provided minimal to no benefits.

### Effects of PKC agonist, in combination with PEP005 or JQ1, on levels of H3K27me3 in latently infected cells

**Figure 3:**
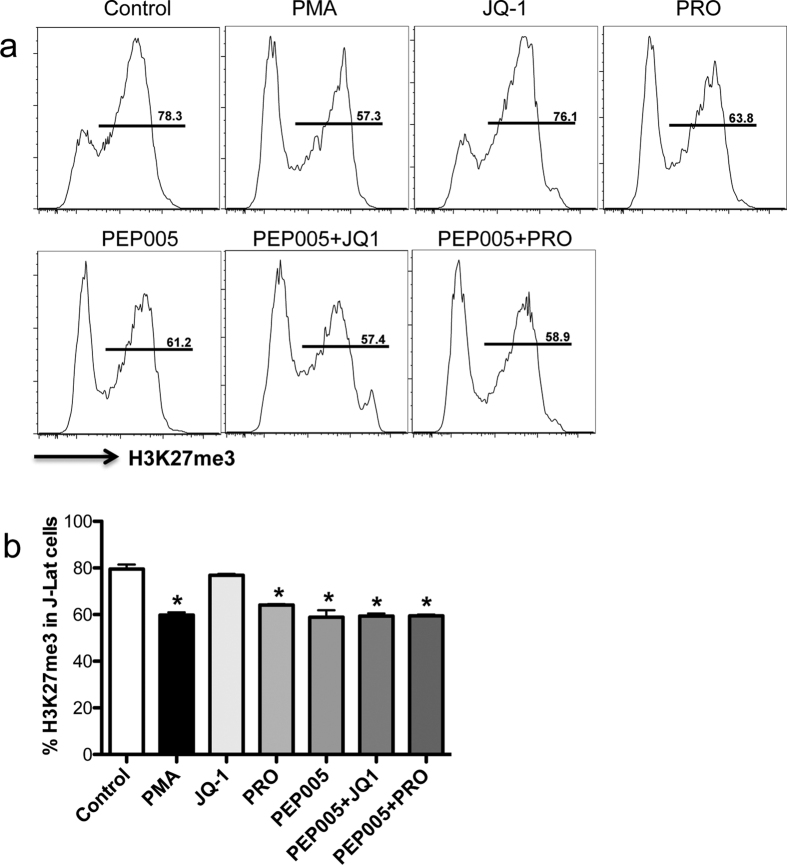
Changes in histone 3 lysine 27 (H3K27) trimethylation in Jat-Lat-Tat-GFP cells treated with compounds alone, and/or in combination *in vitro*. (**a**) Representative histogram of H3K27me3 expression in cell lines treated with compounds alone or combination; (**b**) Statistical analysis of H3K27me3 expression in cell lines treated with compounds alone or combination. These data are representative of at least three independent experiments. *P < 0.05.

Epigenetic histone modification at histone post-translational levels, such as histone 3 lysine 27 (H3K27) trimethylation (H3K27me3), is affiliated with chromosomal condensation and gene repression^55^,^56^. We thus examined the changes of H3K27me3 in compound-treated J-Lat cells. The data showed that PKC agonist treatment alone or in combination could significantly reduce levels of H3K27me3, compared with JQ1 treatment alone or unstimulated controls (Fig. 3a and b). These results indicated that levels of H3K27me3 reflected activation states of latently infected cells, which suggest the PKC agonists promote histone demethylation, cell reactivation, and downstream active transcription.

## Discussion

Combined antiretroviral therapy (cART) is effective in suppressing HIV replication, but the persistence of latently HIV-infected cellular reservoirs remains the major obstacle to virus eradication. Given the need for cure strategies to purge residual viral reservoirs in patients on ART, the search for new potential compounds capable of efficiently reactivating viral latency, is a major priority.

To eliminate viral latency, histone deacetylase inhibitors (HDACi), such as SAHA, are used to reactivate virus latently cells, and to promote virion release and lytic gene expression from an extremely small minority of viral reservoir, which is induced by herpes simplex virus (HSV), human cytomegalovirus (HCMV) or HIV^20^,^23^,^24^,^57^,^58^. However, recent studies indicate SAHA cannot effectively reverse latency in cells from patients on ART, or reduce the size of the latent reservoir, despite effectively inhibiting histone deacetylases^59^,^60^,^61^. SAHA may successfully increase viral transcription, but fails to effectively enhance viral translation^61^. Moreover, SAHA treatment may increase susceptibility of uninfected CD4 T cell to HIV, and impair virus-specific CTLs^62^,^63^. In comparison, synthetic analogues of protein kinase C (PKC) agonists represent HIV latency reversing agents (LRAs) of interest. We thus tested and comprehensively evaluated these PKC agonists for maximal efficiency in inducing cell reactivation, without inducing cytotoxicity *in vitro*.

The effects of these three PKC agonists on cell activation, HIV LTR-driven GFP expression, and cytotoxicity were tested over a broad range of doses and combinations *in vitro*. As indicated in Fig. 1c–e, these three PKC agonists demonstrated different extents of latency reactivation in our assays while maintaining >90% cell survival. Notably, PEP005 induced maximal GFP expression (~45%), compared with prostratin (~38.5%), bryostatin-1 (~28%) and PMA positive controls (~47%). Importantly, the optimal concentration of PEP005 was far lower in inducing maximal HIV latency reactivation (10 ng/ml), compared with prostratin (1 μM or 390 μg/ml) and bryostatin-1 1 μg/ml). With increasing concentrations, the levels of GFP expression were rapidly reduced to 20% in prostratin (5 times of optimal concentration) or byrostatin-1 treatment (5 times of optimal concentration), accompanied with lower cell survival (prostratin, <80%; bryostatin-1, 40%), whereas PEP005 treatment still maintained high levels of GFP expression (>30%) as well as cell survival (>80%) even at 50 times the optimal concentration. These findings indicated that PEP005 possesses high efficiency to reactivate HIV latency with minimal cytotoxicity.

Given JQ-1 could specifically reactivate HIV-infected cells^50^,^51^, JQ1 treatment combined with other PKC agonists may increase specificity of target cells. We tested the ability of JQ1 treatment alone and in combination with PKC agonists here. However, HIV Tat expression is limited in latently HIV-infected cells, and JQ1 treatment alone cannot fully initiate HIV gene expression, as indicated in our results that JQ1 treatment induces minimal GFP expression from cells (~15%, compared to ~10% in controls) (Fig. 1f). In contrast, PKC agonists treatment in combination with JQ1, modestly increased levels of cell activation and reactivation of HIV latency, and reduced histone methylation (Figs 1g and 3), consistent with recent reports showing combined treatment causes release of HIV viruses to levels similar to positive control stimulation in *ex vivo* cultures^45^. However, Jiang *et al*. reports that PEP005, in combination with JQ1, increases reactivation (~*30% GFP*+ *cells*) and increased levels of HIV RNA transcripts in HIV latently infected cells (~260) *in vitro*, compared with PEP005 treatment alone in reactivation (~15%) and relative levels of HIV RNA (~40) by LTR region measurement^32^. This discrepancy may be attributed to differences in data analysis, as it is known that HIV transcripts in infected cells largely correlate with cell activation^64^. For examples, raltegravir treatment reduces T-cell activation, which is higher at baseline in subjects with detectable 2-LTR circles^65^,^66^,^67^. In our experiments, cell reactivation and GFP expression were examined in live cells, as dead cells were excluded by Aqua Live/Dead cell staining. JQ1 plays role in reactivation of HIV latency via HIV Tat expression in HIV-infected cells, relying on state of cell activation. In fact JQ1 treatment alone did not increase, but reduced T cell activation, as indicated by lower levels of CD69/HLA-DR in J-Lat cell or rhesus primary CD4 T cells (Fig. 2). Importantly, the *in vivo* half-life of JQ1 in plasma is only ~1 h (intravenous injection) or 1.4 hrs (oral administration)^68^,^69^, thus limiting its potential for clinical applications. Due to this limitation of JQ1 in HIV latency reactivation, we thus evaluated whether other PKC agonists or combinations could increase reactivation of virus-infected cells. Our data showed that PEP005 could significantly increase GFP expression, cell activation and histone demethylation, similar to PMA stimulation. However, the combination of PEP005 with others did not significantly produce reactivation of HIV latency. These findings suggested that PEP005 alone, showing higher efficiency and lower cytotoxicity, may be the better candidate for HIV latency reactivation and for potential cure strategies in HIV therapy.

## Methods

### Ethics statement

All animals in this study were housed at the Tulane National Primate Research Center in accordance with the Association for Assessment and Accreditation of Laboratory Animal Care International standards. All studies were reviewed and approved by the Tulane University Institutional Animal Care and Use Committee. Animal housing and studies were carried out in strict accordance with the recommendations in the Guide for the Care and Use of Laboratory Animals of the National Institutes of Health (NIH, AAALAC #000594) and with the recommendations of the Weather all report; “The use of non-human primates in research”. All clinical procedures were carried out under the direction of a laboratory animal veterinarian. All procedures were performed under anesthesia using ketamine, and all efforts were made to minimize stress, improve housing conditions, and to provide enrichment opportunities (e.g., objects to manipulate in cage, varied food supplements, foraging and task-oriented feeding methods, interaction with caregivers and research staff).

### Cell culture and treatments *in vitro*

Jurkat-Lat Tat-GFP (A1) T cell line, which is infected with retroviruses containing LTR-Tat-IRES-GFP, and expresses green fluorescence protein (GFP) upon reactivation^53^,^54^, was used to study HIV latency and reactivation in this study. This cell line was obtained from NIH AIDS Reagent Program. For primary CD4 T cells, mononuclear cells from peripheral blood were isolated from chronically SIV-infected rhesus macaques. CD4 T cells were isolated by MACS Pan T-Cell Isolation Kit, biotinylated anti-CD8 (BioLegend) and anti-Biotin microbeads (Miltenyi). The cells were cultured in an RPMI 1640 medium with 10% fetal bovine serum (FBS), 1% of Pen/Strep, and 1% L-Glutamine. The cells were maintained in 37 °C with 5% CO_2_. To test cell reactivation, cells (5 × 10^5^) were treated with a series of concentrations of PEP005 (R&D), Prostratin (R&D), Bryostatin-1 (SIGMA), SAHA (SIGMA), or JQ1 (R&D) in 96-well U-bottom plates for 24 h. The phorbol-12-myristate-13-acetate (PMA, SIGMA) was used as positive control (5 ng/ml). In combination treatments, all compounds were tested using the optimized concentrations to reactivated HIV latency. After stimulation, cells were harvested and analyzed by flow cytometry using Live/Dead cell discrimination/activation markers, and for GFP expression as below.

### Phenotyping

Cells were stained with: CD3 (SP34), CD8 (SK1), CD4 (L200), CD69 (FN50), and HLA-DR (L243). All antibodies and reagents were purchased from BD Biosciences Pharmingen (San Diego, CA) unless otherwise noted. For J-Lat Tat-GFP cell line, cells were stained with surface CD69 and intracellular H3K27me3 (Cell Signaling). Cell viability was analyzed by Aqua Live/Dead cell staining kit (Invitrogen). Stained samples were resuspended in BD Stabilizing Fixative (BD Biosciences) and acquired on a BD FACS Verse flow cytometer (Becton Dickinson). Data was analyzed with Flow Jo software (Tree star, Ashland, OR).

### Statistics

Graphical presentation and statistical analysis of the data were performed using GraphPad Prism 4.0 (GraphPad Software, SanDiego, CA). Comparisons between groups were analyzed by a one-way ANOVA and Mann-Whitney T-test. P values < 0.05 were considered statistically significant.

## Additional Information

**How to cite this article**: Brogdon, J. *et al*. *In vitro* effects of the small-molecule protein kinase C agonists on HIV latency reactivation. *Sci. Rep.* **6**, 39032; doi: 10.1038/srep39032 (2016).

**Publisher's note:** Springer Nature remains neutral with regard to jurisdictional claims in published maps and institutional affiliations.
